# Service user and care giver involvement in mental health system strengthening in Nepal: a qualitative study on barriers and facilitating factors

**DOI:** 10.1186/s13033-017-0139-1

**Published:** 2017-04-19

**Authors:** Dristy Gurung, Nawaraj Upadhyaya, Jananee Magar, Nir Prakash Giri, Charlotte Hanlon, Mark J. D. Jordans

**Affiliations:** 1 Transcultural Psychosocial Organization (TPO) Nepal, Baluwatar, GPO Box 8974, Kathmandu, Nepal; 2 HealthNet TPO, Amsterdam, The Netherlands; 3 Nepal Mental Health Foundation (NMHF), Lazimpat, GPO Box 557, Kathmandu, Nepal; 4 Institute of Psychiatry, Psychology and Neuroscience, Centre for Global Mental Health, King’s College London, London, UK; 5 Department of Psychiatry, School of Medicine, College of Health Sciences, Addis Ababa University, PO 9086, Addis Ababa, Ethiopia; 6 War Child Holland, Helmholtzstraat 61-G, 1098 LE Amsterdam, The Netherlands

**Keywords:** Service user and caregiver, Patient involvement, Stigma, Empowerment, Nepal

## Abstract

**Background:**

Service user and caregiver involvement has become an increasingly common strategy to enhance mental health outcomes, and has been incorporated in the mental healthpolicies of many developed nations. However, this practice is non-existent or fragmented in low and middle income countries (LMICs). Instances of service user and caregiver involvement have been rising slowly in a few LMICs, but are rarely described in the literature. Very little is known about the context of user and caregiver participation in mental health system strengthening processes in a low-income, disaster- and conflict-affected state such as Nepal.

**Methods:**

This study explores (a) the extent and experiences of service user and caregiver involvement in policy making, service planning, monitoring, and research in Nepal; (b) perceived barriers to such involvement; and (c) possible strategies to overcome barriers. Key Informant Interviews (n = 24) were conducted with service users and caregivers who were either affiliated to a mental health organization or receiving menta health care integrated within primary care. Purposive sampling was employed. Data collection was carried out in 2014 in Chitwan and Kathmandu districts of Nepal. Data analysis was carried out in NVivo10 using a framework approach.

**Results:**

The involvement of service users affiliated to mental health organizations in policy development was reported to be ‘tokenistic’. Involvement of caregivers was non-existent. Perceived barriers to greater involvement included lack of awareness, stigma and discrimination, poor economic conditions, the centralized health system, and lack of strong leadership and unity among user organizations. Increased focus on reducing public as well as self-stigma, improved policy frameworks and initiatives, and decentralization of care are some strategies that may facilitate service user and caregiver involvement.

**Conclusion:**

The study highlighted need for user and caregiver networks free from competing interests and priorities. Improved policy frameworks and decentralization of care may support meaningful service user and caregiver involvement.

**Electronic supplementary material:**

The online version of this article (doi:10.1186/s13033-017-0139-1) contains supplementary material, which is available to authorized users.

## Background

Involvement of service users and caregivers, mainly in advocacy, service planning, service monitoring, and research, has been promoted globally as a strategy to help bolster health and quality of life of service users by improving their mental health. It is also advocated as a method to overcome human rights violations, systematic disempowerment and marginalization of persons with mental disabilities [1–3]. The term “user and caregiver involvement” has become a buzzword that has been embedded in the policy and guidelines of many high income countries (HIC) [4–7].

In HICs the service user and caregiver movement stems mostly from dissatisfaction with a paternalistic medical model of care that views patients as passive subjects unable to make their own decisions [8]. Another driver has been the development of consumerist notions of health care that have brought about increased choices and voices for service users [8, 9]. Various theoretical models and frameworks have been used to conceptualize and shape the involvement of service users and caregivers in health services. Arnstein’s “ladder of citizen participation” describes different degrees of participation in terms of different rungs, with citizen control at the highest rung followed by delegated power, partnership, placation, consultation, informing, therapy, and finally manipulation at the lowest [10]. Similarly, Choguill’s ladder of participation in low and middle income countries (LMIC) [11], and Hickey and Kipling’s participation continuum also explore the extent of service user involvement in decision making [12]. Charles and DeMaio offer a three-dimensional framework that describes the key aspects and goals of lay participation in health care decision making [13]. A more recent model of user involvement, proposed by Tritter and McCallum [14], uses the image of mosaic tiles to represent the complex and dynamic relationships between users and other stakeholders in the involvement process.

The meaning and language of user involvement has also evolved and varied over the years. Rogers and Pilgrim described different conceptualizations of users based on their involvement: ‘users as patients’ (a traditional view where users are viewed as passive recipients of services), ‘users as consumers’ (where professionals view users not as objects but as consumers having power to make choices and provide opinions), ‘users as survivors’ (where users campaign collectively for their human rights), and ‘users as service providers’ (where users are involved in service delivery and service development) [15].

While the practicalities of achieving involvement of service users and caregivers are still being deliberated, the importance of such involvement for better mental health care outcomes and responsive health systems is now well recognized [16, 17]. Although limited, previous literatures have highlighted the relevance of service user and caregiver involvement in policy making [6], service planning and delivery [18–20], evaluation [21, 22] and research [23–25]. User and caregiver involvement in mental health is deemed crucial, as service users and caregivers are most able to understand the realities of life of other service users and caregivers, for example, with respect to stigma and discrimination, livelihood challenges and economic constraints [26].

The World Health Organization (WHO), in a seminal report [3], lauded the role of consumer and family movements in positively influencing mental health policies and practices. Such involvement is considered to be especially important in LMICs where weak national mental health systems are pervasive [27, 28]. The importance of user and caregiver involvement was reinforced by the endorsement and ratification of the UN Convention on the Rights of Persons with Disabilities (UNCRPD), which calls for equal and full participation of persons with disabilities, including those with mental health conditions, in treatments and in the development of mental health laws, policies, and programs [29].

Despite such high level endorsements, user and carer involvement in mental health care planning and delivery processes is mostly restricted to HICs [2, 18]. However, even in a country like the UK, where recent policies and health service guidelines necessitates care plans to be formulated in conjunction with the user and carer groups, it is failing to be realized in practice to the satisfaction of carer and user groups [30, 31]. In LMICs, such carer and service user involvement is mostly non-existent or weak and fragmented [32, 33]. Instances of service user and caregiver participation have increased over the years in LMIC with the formation of users’ networks, groups, and organizations, as is seen in some African nations such as Uganda, Zambia, Kenya, and Tanzania [34]. However, initiatives of such groups are rarely described in the literature [35]. There are very few studies that delineate the extent and modes of such involvement in LMIC, and even though service user and caregiver involvement is touted by many governments and organizations, examples of best practices remain scattered. As a consequence, very little is known about effective ways to promote service user and caregiver involvement in mental health policy-making, planning, service monitoring, research and evaluation.

In Nepal, service user involvement, mainly in mental health promotion and advocacy, has recently received a boost through the establishment of national level service user organizations [36] and through the signing of UNCRPD and its optional protocols by Nepal in 2008 [37]. However, the experiences of service users and caregivers with respect to involvement, their achievements and struggles, and their views regarding effective ways to promote service user and caregiver involvement remain undocumented. This paper aims to assess the experiences and challenges of service users and caregivers regarding their involvement in mental health system strengthening processes in Nepal, including policy development, service planning, monitoring, and research, and to identify effective ways to enhance service user and caregiver involvement in system level processes.

## Methods

### Background and setting

This qualitative study is part of the Emerald programme (Emerging mental health systems in low and middle-income countries) that is being carried out in six countries: Ethiopia, India, Nepal, Nigeria, South Africa, and Uganda. Emerald seeks to identify key health system barriers to, and solutions for, the scaled-up delivery of mental health services in LMIC, and by doing so improve mental health outcomes in a fair and efficient way. One of the thematic areas of the project is to empower, equip and facilitate the involvement of service users and their caregivers to support mental health system strengthening [38].

This study was carried out in the Kathmandu and Chitwan districts of Nepal. Service users and caregivers affiliated to user and caregiver organizations were mostly based in Kathmandu. Chitwan district was selected as a second site in order to understand the perspective of grassroots level mental health service users and caregivers from rural populations who have no affiliation to user networks or organizations. In Kathmandu district, participants for the study were approached through service user or caregiver organizations. In Chitwan district however, service users and their family members seeking mental health services at primary health care facilities as part of the Programme for Improving Mental Health carE (PRIME) [39, 40] were recruited. The PRIME project aims to investigate the implementation and scaling-up of mental health care services into primary health care setting in five countries including Nepal. In Nepal, the project is being implemented by TPO Nepal, which is also the implementing organization for the Emerald project. As no mental health user groups or organizations exist in Chitwan district, grassroots level service users and caregivers were recruited through the PRIME project.

### Sampling and data collection

Our study applied purposive sampling. A total of 24 key informant interviews were conducted, with the sample comprising service users who are referred as self-advocates affiliated to mental health organizations (n = 7), caregiver representative of a mental health organization (n = 1), mental health service users not affiliated to any organizations (n = 7), and the latter’s caregivers (n = 9). Seven participants were from Chitwan (4 males and 3 females) and 17 were from Kathmandu (13 males and 4 females). Nine study participants (37.5%) reported using mental health services (medicine and/or counseling) for 5 or more years, 21% of the participants used services for 1–5 years, 25% for less than a year while remaining (16.5%) declined to provide the information.

Data collection was carried out in 2014. Prior to the data collection, the research team (comprising of 6 researchers; 2 males and 4 females) received a one week training that included familiarization with the research design, research objectives and ethics, translation of the topic guides from English to Nepali, contextualization into Nepali culture, and interview role-plays. All the researchers involved in the data collection had more than 2 years of experience of conducting mental health research and were aware about nuances of interacting with individuals with mental health problems. To deal with any gender or cultural issues, the female participants were interviewed by female researchers while male participants were interviewed by males.

Semi-structured Key Informant Interviews (KIIs) were conducted in Nepali language. The interviews were conducted in the organizations where the mental health user advocates were affiliated in Kathmandu. In Chitwan district, the interviews were conducted at homes and health facilities (as suggested by the participant themselves). The Interview schedule consisted of demographic information and a topic guide that covered service user and caregiver involvement in four major areas of system strengthening processes: (a) policy making, (b) service planning and development, (c) monitoring, and (d) research. Interview guide used during the study is available as Additional file 1. Researchers were mobilized in pairs to conduct face-to-face interviews. Responses were mostly audio-recorded and, in cases where consent for audiotaping was not given, responses were noted manually. Participants were recruited in the study until no new additional information were emerging from the interviews. The researchers involved in the data collection process convened regularly to reflect on the nature and quality of information generated and if data saturation was achieved.

### Data management and analysis

A framework approach was used for the analysis of the data [41]. The interviews obtained through KIIs were first transcribed. The transcribed data, along with the manually recorded notes, were then translated into English by professional translators who had previous experiences of working in mental health area. A portion of the translated data (10%) was crosschecked against the original by the research supervisor (NU) who is fluent in both Nepali and English language.

For the development of a coding framework, two researchers first read and coded 40% of the data separately and identified an initial set of codes and emergent themes. These two separate sets of codes and themes were then compared with each other and discussed among the research team. Based on the discussion, a coding framework was generated, discussed, and finalized. QSR NVivo 10 software was used for indexing and charting of the data. The coding was carried out by two researchers and they met regularly to discuss any new issues and possible solutions to the interpretation of pre-existing codes. Coding comparisons were done based on the background characteristics of the research participants. Findings were compared between carers vs service users, service user advocates vs grassroots service user and males vs females. Any deviant information generated from coding comparison was reflected in the reporting of the findings. To check for the validity of the findings from the study, the final framework matrix generated after coding and analysis was discussed with the entire research team and with mental health survivor and advocate (NPG) to seek feedback on the emergent themes and descriptions.

## Results

### Current situation of service users and caregivers’ involvement

Participants indicated limited involvement of service users in policymaking processes and almost non-existent involvement in other areas of national health system processes (i.e. planning and service development, monitoring, and research). The concept of having representation in policy making processes was absent among caregivers. Similarly, involvement in such processes seemed a foreign idea to many of the service user participants, especially among those living in rural areas. They did not understand the meaning of mental health planning and policies, and were unaware of how or why such processes took place. Most participants however, showed interest in taking part in such processes if given opportunity and training.

Any involvement of service users in policy level processes was limited to those affiliated to service user organizations in Kathmandu. However, they too reported that they were being ignored in important decision-making processes most of the time.

*“Although there are many programs being organized for policy making, we are not invited. They know we exist, but they don’t invite. There are few of us and our voice is not heard. We don’t get to participate in the policy drafting.” (Service user organization representative, Kathmandu, 34, Female)*

The service users who were involved in some of the government policy programs viewed the quality of their involvement as poor. These service users mentioned that although they were invited to group discussions and consultations, they did not have any direct involvement in decision making and reported dissatisfaction with this degree of participation.

*“Yes, although they invite us, they don’t make us fully participate. For instance, during the drafting of the latest mental health bill it was said that there was some participation of service users. But they never gave those participants chance to express their view. They only used their name. That’s why I am saying there is no meaningful participation” (Service user organization representative, Kathmandu, 35, Female).*

A service user expressed that such tokenistic involvement had caused service users to be treated as “non-essentials” by the government, as well as by Non-Governmental Organisations (NGOs),

*“It is [* 30 *] use and throw concept, where service users are called to participate showing token benefits and once they come to the program, they are disregarded. There is no sustainable enrollment.” (Service user organization representative, Kathmandu, 33, Male).*

In terms of involvement in monitoring of mental health services, the participants voiced that they had no idea about monitoring mechanisms of the government for mental health and, even if such mechanisms did exist, they were not involved. With regard to research, some participants shared that their experience of involvement was limited to that of being research participants. They complained that they were not contacted again after the data collection process and so were not aware how the data were used.

### Barriers to involvement of service users and caregivers

#### Awareness and information

Lack of education and awareness, compounded by poor economic conditions and being from a rural region, were cited as barriers that hindered users and caregivers’ involvement in system strengthening processes. Participants mentioned that these factors lowered their confidence and increased their feeling of inferiority.

*“It’s scary for a person from a village to go to the city. I think people who live in the city are clever. I fear they will shout at us without really listening.” (Non*-*affiliated service user, Chitwan, 55, Female)*

*“An uneducated person won’t know anything. The educated person can talk and contribute. They [policy makers] will listen to the things said by the educated people.” (Non*-*affiliated service user, Chitwan, 45, Female)*

While this was more apparent among study participants from rural parts, where the majority did not have higher education, it was also cited as a barrier by educated participants, as they did not know where to go or whom to meet. They felt that they were ill-equipped to participate in such processes due to lack of technical knowledge about processes relating to policy making, monitoring and evaluation, research designs, and international laws.

Lack of awareness amongst policy makers themselves was cited as a distinct barrier to involvement. Participants suggested that because policy makers are uninformed about mental health issues and the need for service user and caregiver involvement, they are unable to prioritize mental health in government health strategies or initiate any policy processes to aid user and caregiver involvement. In addition, lack of awareness was also cited as the main driver of stigma towards service users among policy makers.

#### Stigma and discrimination

For many study participants, their desire to participate in system level processes was overshadowed by their fear of stigma and discrimination. They mentioned that there was no incentive to identify themselves or work in this sector; instead of ‘glory’ or ‘support’, they reported receiving only stigma and discrimination. Due to the presence of stigma in the community, the study participants argued that service users and caregivers feared identifying themselves as people with mental disability.

*“They don’t come out due to stigma! How would you identify them? How would you bring them in front? To be honest, these family members of the service users don’t want to be involved at all due to stigma. They say that they don’t know about such problems. They don’t want to get attached to this issue. Due to the stigma, the whole family becomes humiliated and so I don’t see the family members coming forward.”* (Service user organization representative, Kathmandu, 32, Male)

The paucity of involvement of service users was also attributed to the stigmatizing attitudes of government and health personnel in the predominantly biomedical health care model. Service users claimed that the government mostly involved psychiatrists to make key policy decisions and felt reluctant to involve service users because psychiatrists viewed service users as their patients and not as equal partners capable enough to work together on the same level:

*“There must be some ego issues regarding the power. Or they don’t want to change their orthodox view thinking we can’t do anything. They don’t see the patient as person.” (Service user organization representative, Kathmandu, 35, Female)*

Although the government in its recent initiatives acknowledged the need to involve service users, the study participants remarked that service providers and policy makers refused to do so on equal grounds.

*“The service providers want to form policies and develop services and seek our token support. They are the ones to decide whether they want to involve us or not. They expect our support but they want the power [to make the decision] too. If they can’t create the environment where people sit on equal ground and look straight at each other, then my argument is for whom are they developing their services and programs? " (Service user organization representative, Kathmandu, 33, Male).*

#### Economic condition and competing priorities

Due to poor economic conditions among service users and caregivers, their first priority was to meet the basic needs of the family. Their need, therefore, was for free treatments and medicines, and income generating activities, rather than involvement in system strengthening processes. Participants mentioned that they had to earn their living and so did not have time or money to spare to go to the cities where most of the system level processes took place.

#### Centralized national health system processes

Centralization of health system processes was recognized as one the factors limiting access to participation, as most service users live in rural areas while such processes take place in cities such as Kathmandu.

*“If I am the service user at the grassroots level, I don’t even have the access to the district development level. How can a service user like me have access to the national level?”* (Service user organization representative, Kathmandu, 35, Female).

Some participants reported that the appointment of the Director for the only mental hospital in country as the de facto focal point for mental health by the Ministry of Health and Population (MoHP) and lack of district level mechanisms for mental health had resulted in most of the government’s initiatives taking place in Kathmandu. The aspirations of service users and the principle of the centralized mental hospital were at odds, making it difficult for service users to form good working relationships with the mental hospital.

*“It is very difficult to build ‘working together relation’ with Mental Hospital in our country. It is because community based mental health does not agree with some of the things in the management of Mental Hospital. So, there is no working together relation.”* (Service user organization representative, Kathmandu, 32, Male)

#### Leadership and unity

The study participants acknowledged that they lacked strong leadership and unity in the service user and caregiver community in Nepal. This was perceived to have inhibited the possibilities of involvement in health system processes, since a collective voice cannot be heard and collaborative work is not common. The disjuncture among service users, especially among those representing different organizations, has allegedly led to disagreement among the small number of service user advocates.

*“…Most organizations think that they are the pioneer organizations in this field and everyone should listen to them …The service users working as advocates feel that they are the only ‘survivors’ and they should be [present] everywhere…They don’t respect each other and they cannot come together.”* (Service user organization representative, Kathmandu, 44, Male)

Some participants hinted that a sense of competition among service user representatives and advocates has led to some of the upcoming service user advocates being ignored by those already established in the field.

*“Our organization is not fully established. We are opening a new organization and we need support….But we do not have any knowledge regarding these things. I personally feel that since I started this organization, we have been ignored. We have been told by few others in this field that other organizations are marginalizing us. I even didn’t know about the issue of the new policy. I knew only after someone told me that the new policy has been circulated.” (Service user organization representative, Kathmandu, 33, Female)*

### Strategies for increased involvement

#### Raising awareness

The study participants claimed that improved awareness among service users, caregivers, policy makers, and the general population is needed to foster a suitable environment for their involvement. As mentioned in Table 1, involvement of media was thought to be an important method to educate people in the community. Some of the study participants suggested that incorporating mental health topics in school education would raise awareness in a cost-effective manner. Some also recommended personal means of raising awareness such as interpersonal interactions with neighbors and friends that would help build friendship and promote support among service users.

**Table 1:** Summary of barriers to involvement and strategies to facilitate involvement

Barriers hindering involvement in national health system processes	Strategies to facilitate involvement
*Lack of awareness and information* Lack of awareness regarding the ‘why’ and ‘how’ of involvement among service users, caregivers, and policy makers Lack of confidence to participate due to lack of information Ignorance among policy makers that leads to failure to prioritize mental health	*Raising awareness* Use of media: documentary/drama, street-plays, posters, pamphlets, radio, TV programs, billboards Incorporation of mental health issues in school education Interpersonal interactions among community members For policy makers- interaction with service users/caregivers, field visits to health centers, awareness workshops
*Stigma and discrimination* Service users and caregivers feel humiliated and don’t want to identify themselves or become involved due to stigma No space in government positions for service users Psychiatrists unwilling to work with service users on equal grounds	*Reduction of stigma* Through awareness-raising, education, employment opportunities, quota for government positions Getting rid of discriminatory words such as ‘service users’ and ‘service providers’
*Poor economic conditions and competing priorities* Focus on earning a living, so no time to spare for involvement Expectation of free treatments and medicine, involvement in income generating activities rather than system processes	*Formation of service user and caregiver groups at grassroots level* Bottom-up approach: service user/caregiver groups should be established in villages Supports involvement of service users/caregivers from rural areas
*Centralization of national health system processes* No access to system strengthening processes for those living in rural areas; health system processes mostly take place in major cities	*Capacity building* Training should be conducted by the government Training should address basic knowledge of mental illness, its types, and treatments, mental health systems and system strengthening, their needs and roles of service users/caregivers
*Lack of strong leadership and unity among service user community* Disjuncture among service users representing organizations A sense of competition among service user organizations Conflicting views regarding selection of representatives Lack of consensus on how/to what extent service users should be involved in policy development	
	*Selection of representatives* Selection to represent the population from grassroots level Representation of all demographic, economic and geographical groups needed
	*Methods of involvement* Involvement should take place at different levels of policy making Monitoring: formation of monitoring committee with service users, caregivers, service providers, government employees as its members Research: involvement mainly in data collection

The participants argued that changing of attitudes was essential for government officers and policy makers as well. They thus proposed awareness classes, field visits (to mental health clinics), and interaction with service users and caregivers to foster greater understanding.

#### Reduction of stigma and discrimination

The majority of study participants focused on reduction of stigma and discrimination as a major strategy to facilitate user and caregiver involvement. Raising awareness in the community and at the government level was a recurring theme in comments on how to deal with stigma, along with education and employment opportunities for service users, and opportunities to apply to government level positions.

Another means of reducing stigma mentioned by some service user representatives and advocates was getting rid of the concept of “service user” and “service provider,” as these terms- as well as their equivalent Nepali words ‘*sewa grahi’* and *‘sewa pradayak’*- induced a sense of hierarchy and prevented doctors and patients from standing on equal ground. They proposed that ‘survivors’ or ‘advocates’ would be a more fitting term than‘service users’.

*“The term of service users alone is stigmatizing. There is a notion of hierarchy in it with the provider as an authority. The identity that you give them after a couple of interactions makes them detached with you. This is the reason why the drop*-*out rate is high after certain time.” (Service user organization representative, Kathmandu, 33, Male)*

#### Formation of service user and caregiver groups at the grassroots level

Building close-knit service user and caregiver groups in the community was encouraged, as it was assumed to serve as self-help groups, work as advocacy groups, and help to identify representatives for involvement in system strengthening processes. Some caregivers believed that through such support groups, even those residing in rural areas could be involved in system strengthening processes. Participants suggested that the grassroots organizations and the government could conduct meetings at the local level to consult with such groups regarding their issues, views, and suggestions pertaining to mental health plans and policies. They suggested that this would help service user and caregiver groups to contact their local political party members or parliament members and communicate their grievances, issues, and concerns so that these could be relayed to policy makers and government officials at central level.

#### Capacity building and training

It was emphasized that the best possible way of building capacity of service users and caregivers was for the government to conduct training. Service users complained that although they had participated in training programs, none of the training was conducted by the government. Therefore, participants recommended that such training be conducted by the government using its own training tools, in order to make the government more accountable, and the training, more sustainable. The areas for capacity building and training underscored by study participants are highlighted in Table 1.

#### Representativeness in selection

There was a lack of consensus among study participants regarding selection of service user representatives. Some study participants, mainly caregivers and non-affiliated service users from Chitwan district, mentioned that those with chronic or severe mental illness would not be able to participate and so those having mild form of illness such as general anxiety and depression should be selected as representatives. However, service users working as advocates claimed that there should be representation of all types of mental illness as different types of mental illness are associated with different issues.

Knowledge and experience regarding the system strengthening process were also set as key criteria for selection of representatives. Study participants put forward strong views regarding representation of all demographic, economic, and geographical subgroups of service users/caregivers, particularly poor people from rural areas. For example, one caregiver cautioned,

*“The one who is from the rich family perceives mental health differently than the one whose economic condition is low, and the one who cannot afford for his medicines. The issues of both the groups should be addressed properly and representation should be done from all sectors… We should not involve only selected service users whom we already know. Representation of all should be there.”* (Non-affiliated caregiver, Kathmandu, 28, Male)

#### Methods of involvement

Most service users affiliated to an organization argued that greater involvement in drafting of policy and their inclusion in drafting committees was a must to make their participation more meaningful. Some caregivers and service users, however, differed in their opinions. They believed that service users and caregivers should be involved, but not directly:

*“Rather than involving them directly in the policy making, what we can do is, in a loose forum we can discuss a questionnaire which can address their problems. They can give their view on it. Service users won’t be making the policy but at least we can take their view in the policy making. We can take loose data from them and ‘concretely and strategically’ develop the data into policy.”* (Non-affiliated service user, Kathmandu, 29, M)

Some conceived that service users should be involved only in later phases because a working committee (consisting of individuals selected to draft policies) should have ‘expert stakeholders’ who are experienced, while participants from rural community believed that policymaking was the role of the government and policymakers, and that they should be the ones making the policies instead of service users and caregivers.

## Discussion

This study set out to explore experiences of service user and caregiver involvement in national mental health system strengthening processes and assess the barriers to, and strategies for, facilitating such involvement. The findings of this study show limited involvement of service users and caregivers in policymaking and health system strengthening processes in Nepal. Framed within Arnstein’s ladder of involvement, in policymaking, service users were *informed* and/or *consulted* on the draft policies. Service user involvement in Nepal, although present, is mainly limited to service users affiliated to organizations.

For caregivers, involvement in national mental health system strengthening is simply non-existent. This could be because both service providers and service users consider the caregiver’s role to be ‘less important and realized’ [42]. Nevertheless, the importance of caregiver involvement has been highlighted [3, 17, 43]. Caregivers are a vital part of the service system and often have different viewpoints from those of service providers and service users [44]. A study conducted in Iran showed that caregivers could become effective case managers when provided with training [45]. This study also shows the need for governmental and non-governmental organizations to take the initiative in promoting caregiver involvement in health system processes along with that of service users in Nepal.

Levels of involvement varied among non-affiliated service users and users affiliated to mental health organizations. The service users affiliated to mental health organizations, who also tended to be based in the capital city, reported more experiences of involvement compared to non-affiliated service users from rural areas. Building user groups and networks at village and district levels, as suggested by study participants, could be one strategy to involve other interested service users. Studies conducted in other countries also show that building user and caregiver group networks is necessary to support collective user action (for example, see [6, 14, 46]). However, some study participants warned against *‘NGO*-*isation’* of such groups and networks as this might alter the objectives and priorities of the organizations to compete in the donor driven market. The limited number of service user organizations, mostly concentrated around major cities and vying for increased funding and recognition, appeared to lead to narrow representation of service users and their issues at the grassroots level. This may be one of the reasons underlying the sense of mistrust and reservations towards NGOs repeatedly expressed by our study participants. Although NGOs have played a pivotal role in advocating for the rights of service users in mental health and lobbying for their involvement, the lack of consensus among NGOs has been identified as a key challenge to development of the mental health sector in Nepal [47]. Hayward and Cutler [48] also warn that competition and mistrust among NGOs is a major barrier to grassroots civil society. In contrast, grassroots groups such as self-help groups, with broader representation from mental health service users and caregivers, have been shown to foster empowerment, reduce stigma, and create a favorable environment to advocate for the rights of service users [49].

Among the most recurrent barriers to involvement mentioned were low economic status and lack of awareness, information, and education. The majority of study participants from rural areas mentioned that economic constraints and accompanying time constraints were major barriers to becoming involved or to being interested in participating in system strengthening processes. Our participants’ demand for financial incentives resonates with findings of McDaid’s study [50], which indicated that disparities in economic resources between service users and other participants in mental health policy and planning processes was one of the major barriers to equal participation of users. This suggests organizations and government that plan to engage service users and caregivers may need to provide stipends or other forms of economic support [51–53].

Internalized self-stigma and within-group stigma among the service users and caregivers was also reported by participants in our study. Some believed that users with severe mental illness would not be able to participate and hence should not be selected as representatives, and some even stated that they themselves would not be able to contribute much in the system strengthening processes due to their illness. This may be, to some extent, due to self-stigma among service users, although this was not overtly expressed. Self-stigma has been linked with lower levels of empowerment and fear of disclosure among service users [54]. Nepal’s government has tried to promote inclusiveness and tackle stigma through the creation of a disability quota for government positions. However, due to stigma, persons with mental disabilities fear disclosing their disability, and thus are often unable to compete for employment through the disability quota. This situation illustrates the complexity of confronting stigma and discrimination; in the anti-stigma programs of the government and mental health organizations, it is essential to address not only public stigma but also self-stigma. Highlighting the stories of service users who have disclosed their mental health problems and giving examples of their roles in system strengthening processes might be one effective strategy. Diversity and representativeness in service user involvement is a long standing issue that has been discussed in previous studies [18, 55]. Our findings too indicate the need for increased diversity among service user representatives in terms of types of illness and demography. Decentralization of mental health services and promotion of community-based care could provide additional opportunities for service users from diverse demographics to participate in local processes. Community participation in decision-making, user empowerment and self-help, and a focus on public needs have been emphasized as characteristics of community based care [56]. With the decentralization of power to communities, the voices of different community groups can be heard and a collective agency can be established to deal with problems [27]. Similarly, integration of mental health into primary health care systems could also ensure some level of representativeness at local level by involving service users in the existing management committees of the primary health care centers. As integration of mental health in the public health system is being initiated in some districts by NGOs and government [36], it is timely to consider ways of adapting approaches to service user/caregiver involvement to facilitate effective integration and strengthen the national mental health system.

Government initiative to reform policies was mentioned as another strategy to support service user and caregiver participation. Existing national documents on mental health refer to partnership among the government, International NGOs, and researchers, but do not mention involvement of service users and caregivers [57, 58]. Many of our study participants complained that they had been invited to participate in trainings and research programs run by NGOs, but never in government-led programs. This lack of governmental initiatives to involve service users and caregivers and exclusion of this issue in policy documents contrasts with health sectors such as human immuno-deficiency virus (HIV)/acquired immune deficiency syndrome (AIDS) and tuberculosis, where service users are considered to be important actors of system development, and their inclusion is not only emphasised in policy documents, but also practiced [59, 60]. This could be a result of high prioritization of these problems by the government in its national health strategy, and due to the existence of dedicated centers for these problems in the MoHP. Mental health, by contrast, lacks a separate unit in the MoHP, and there is ambiguity in the appointment of a focal point for mental health related activities, due to which any effort to coordinate with the government has resulted in confusion among mental health civil societies [36]. The establishment of a coordinating body has been suggested as a way to facilitate the role of mental health NGOs in Nepal [47], and this may also help service users and caregivers to raise their issues directly with the government.

## Limitations

Findings of this exploratory study reflect the views of a relatively small group of service users and caregivers and therefore may not be representative of the entire user and caregiver population of the country. This is mainly because there are very few service user organizations and caregiver organizations, and a limited number of user and caregiver advocates working in Nepal. Use of convenience sampling may have led to participants who were more outspoken and easily accessible being represented in the study. This study also addresses the issue of service users and caregiver involvement in system strengthening processes in Nepal through the perspective of service users and caregivers only; to provide a complete picture of the issue, further inquiry is necessary with multiple stakeholders including policy makers and service providers.

## Conclusion

This study shows that meaningful involvement of service users in Nepal is lacking, while involvement of caregivers is simply non-existent. Among service users, experiences and attitudes towards involvement varied between those who were non-affiliated service users and those affiliated to mental health organizations. Self-stigma and within-group stigma, although not mentioned explicitly, was a recurrent theme in the data collected and is one of the major barriers to their involvement. Establishment of user and caregiver networks free from competing interests and priorities (such as those faced by NGOs) was underscored as a strategy to enhance involvement. Improved policy frameworks and improved initiatives (such as those that have been implemented for HIV/AIDS in Nepal) and decentralization of care may support meaningful service user and caregiver involvement.

## Additional file

**Additional file 1.** Interview guide for service user and caregiver involvement in mental health systems processes.

## Abbreviations

AIDS: acquired immune deficiency syndrome
Emerald: emerging mental health systems in low and middle-income countries
HIC: high income countries
HIV: human immuno-deficiency virus
KIIs: key informant interviews
LMIC: low and middle income countries
MoHP: Ministry of Health and Population
NGOs: Non-Governmental Organisations
NHRC: Nepal Health Research Council
PRIME: Programme for Improving Mental Health carE
PNM RESC: Psychiatry, Nursing and Midwifery Research Ethics Subcommittees
TPO: Transcultural Psychosocial Organisation
UNCRPD: United Nation Convention on the Rights of Persons with Disabilities
WHO: World Health Organization
