# Supplementary material for: Service user and care giver involvement in mental health system strengthening in Nepal: a qualitative study on barriers and facilitating factors
Source: Int J Ment Health Syst. 2017 Apr 19;11:30. doi: 10.1186/s13033-017-0139-1 (PMC5395761; doi:10.1186/s13033-017-0139-1)
Supplement: Supplementary file 1 — Additional file 1. Interview guide for service user and caregiver involvement in mental health systems processes. [file 13033_2017_139_MOESM1_ESM.pdf]

Instrument (in English): Service user participation in mental health system strengthening

SN	Questions	Codes	Skip
1.	Name of the respondent	-----------------------	
2.	What is the name of your organization?	-----------------------	
3.	What is your Age	(mention the age)------------------	
4.	Sex	Male ---------------------------------1<br>Female-------------------------------2	
5.	Education	Illiterate------------------------------1<br>Literate-------------------------------2<br>Literate through informal Education---3<br>Literate through Formal Education-----4<br>Standard of education ----------- (mention)	
6.	Occupation	----------------------	
7.	Religion	Hindu---------------1<br>Buddhist------------2<br>Christian-------------3<br>Muslim--------------4	
8.	Ethnicity	Brahmin/Chetri-------1<br>Janjati-------------------2<br>Dalit---------------------3<br>Others------------------(Mention)	
9.	Are you just the representative of this organization or are you a service user as well?	Only organization representative--------1<br>Organization representative and service user---------------2<br>Only service user------3 →	Skip to question 14
10.	How are you involved in this organization?	Founder--------------1<br>Board member-------2<br>Member of management committee-----3<br>Staff--4<br>Others(mention)----------------	
11.	Is this a national or a district level organization?	National Level Organization---------1<br>District Level Organization------------2	
12.	Are you a service user or a care giver organization?	Service User organiztion---------------1<br>Care giver organization-----------------2	
13.	What kind of disorder do your organization represent?	Schizophrenia----------------------------1<br>Bipolar Disorder-------------------------2<br>Depression-------------------------------3<br>Epilepsy-----------------------------------4<br>Alcohol/Drug use disorders-------------5<br>Any mental disorder----------------------6<br>Other (specify)_________________	Skip to the Part A of Interview schedule
14.	What kind of mental health condition do you have?	Schizophrenia----------------------------1<br>Bipolar Disorder-------------------------2<br>Depression-------------------------------3<br>Epilepsy-----------------------------------4<br>Alcohol/Drug use disorders-------------5<br>Any mental disorder----------------------6<br>Other (specify)_________________	
15.	Length of time in contact with services	Medical----------- (years)-----------<br>(months)<br>Counseling --------- (years)--------	

		(months)	

Interview schedule

A. Mental health policy-making

(1) What efforts have you or your organization made towards the development of mental health related policy in Nepal?

   a. Can you tell me about that experience?
   b. What was your contribution?
   c. Why were you selected?
   d. What was your experience? (positive/negative)
   e. What could have improved the experience?
   f. Would training or support in mental health policy-making be useful to facilitate your contribution?
   g. What was the outcome of your involvement?

(2) Do you think service users / service user organizations can contribute to mental health policy-making?
   a. What can and should you or your organization do to contribute towards realistic mental health policy making? (Probe: Where and how)
   b. How would service users be selected to participate?
   c. Would they need training? If so, what type of training would be helpful?
   d. Would they need support? If so, what type of support would be helpful?

(3) What barriers could there be to service user and service user organization involvement in policy-making?
   a. On the side of the service user and service user organization?
   b. On the side of the policy-makers?

B. Mental health planning and service development
Now I am going to ask you about the planning and development of mental health services. For example, the decisions that are made about what type of service is going to be delivered, where it will be delivered, who will deliver it and how they will deliver it.

(4) Have you/your organization ever been involved in any aspects of the design and development of mental health services provided by government/non-government organizations?
   a. **If so,** can you tell me about that experience?
      i. Which mental health service planning were you involved in?
      ii. What was your level of involvement?
      iii. What contribution do you think you made?
      iv. How was the experience for you?(Positive or negative)

v. How do you think other participants viewed your contribution? (Positive or negative) Probe, why?
vi. Is there anything about the process that could be improved?
vii. Do you think any support/ training would be helpful for you to contribute more? If so, what kind of support/training?
viii. What factors would be helpful for you to contribute more in such process?

b. **If not**, what do you think about the idea of service users getting involved in service development in that way?
   i. Would you or your organization be interested to contribute in the mental health service development?
   ii. What do you think could be a benefit of your/your organization's involvement?
   iii. What could be the problems or challenges if you or your organization were to be involved?
      - From your/your organization side?
      - From the side of the service planners?
   iv. Would training be useful to help you contribute more? If so, what kind of training?
   v. What else might help to facilitate greater involvement of patients in this process?

C. Monitoring mental health services

Now I am going to ask you about patient involvement in monitoring the quality of mental health services.

(5) Have you / your organization ever been involved in monitoring the quality of a mental health service provided by government/non-government organizations?

a. **If so**, can you tell me about the experience?
   i. What type of monitoring of mental health service were you involved in?
   ii. What was your level of involvement?
   iii. What contribution do you think you made?
   iv. How do you think other participants viewed your contribution? (Positive or negative) Probe why?

   v. Did you face any kind of problems/challenges during the involvement? If yes, then what kind of problems?
   vi. Do you think you were able to help improve the quality of the service?
   vii. Is there anything about the process that could be improved? If yes, then what are they? Why?
   viii. Would any support/training be helpful for you to contribute more? If so, what kind of support/training?

b. **If not**, what do you think about the idea of involving service users in improving the quality of mental health services?
   i. Would you be interested to contribute in the monitoring of mental health services?
   ii. What do you think could be a benefit of your involvement?
   iii. Do you think there would be any problems or challenges to getting involved?

- From your or your organization's side?
- From the side of the service providers?

iv. Would training be useful to help you contribute more? If so, what kind of training?
v. What else might help to facilitate greater involvement of patients in this process?

D. Research evaluation of mental health services

Lastly, I am going to ask you about patient involvement in research projects to evaluate mental health services.

(6) Have you / your organization ever been involved in a research project to evaluate a mental health service provided by government/non-government organizations?

a. **If so**, can you tell me about the experience?

i. What type of evaluation of mental health service were you involved in?
ii. What was your level of involvement?
iii. What contribution do you think you made?
iv. How do you think other participants viewed your contribution? (Positive or negative) Probe why?
v. Did you face any kind of problems/challenges during the involvement? If yes, then what kind of problems?
vi. Is there anything about the process that could be improved? If yes, what are they? Why?
vii. Would any support/training be helpful to help you contribute more? If so, what kind of support/training?

b. If not, what do you think about the idea of involving patients in research projects to evaluate mental health services?

i. Would you be interested to contribute in the evaluation of mental health services?
ii. What do you think could be the benefit of your involvement?
iii. Do you think there would be any problems or challenges to getting involved?
   - From your/your organization's side?
   - From the side of the researchers?
iv. Would training be useful to help you contribute more? If so, what kind of training?
v. What else might help to facilitate greater involvement of patients in this process?
